# Insomnia symptoms during the covid-19 pandemic: a case-control study

**DOI:** 10.11606/s1518-8787.2023057004801

**Published:** 2023-05-11

**Authors:** Andrea Bacelar, Silvia Gonçalves Conway, Márcia Assis, Victor Menezes Silva, Pedro Rodrigues Genta, Daniela Vianna Pachito, Almir Ribeiro Tavares, Danilo Anunciatto Sguillar, Gustavo Antonio Moreira, Luciano Ferreira Drager, Claudia Roberta de Castro Moreno

**Affiliations:** I Clínica Neurológica Carlos Bacelar Rio de Janeiro RJ Brasil Clínica Neurológica Carlos Bacelar. Rio de Janeiro, RJ, Brasil; II Associação Brasileira do Sono São Paulo SP Brasil Associação Brasileira do Sono. São Paulo, SP, Brasil; III Universidade de São Paulo Faculdade de Medicina Departamento de Psiquiatria São Paulo SP Brasil Universidade de São Paulo. Faculdade de Medicina. Departamento de Psiquiatria. São Paulo, SP, Brasil; IV Akasa - Formação e Conhecimento São Paulo SP Brasil Akasa - Formação e Conhecimento. São Paulo, SP, Brasil; V Hospital São Lucas Clínica do Sono de Curitiba Curitiba PR Brasil Hospital São Lucas. Clínica do Sono de Curitiba. Curitiba, PR, Brasil; VI Universidade de São Paulo Faculdade de Saúde Pública Departamento de Saúde, Ciclos de Vida e Sociedade São Paulo SP Brasil Universidade de São Paulo. Faculdade de Saúde Pública. Departamento de Saúde, Ciclos de Vida e Sociedade.São Paulo, SP, Brasil; VII Universidade de São Paulo Hospital das Clínicas Faculdade de Medicina São Paulo SP Brasil Universidade de São Paulo. Hospital das Clínicas da Faculdade de Medicina. Instituto do Coração. Laboratório do Sono. São Paulo, SP, Brasil; VIII Fundação Getúlio Vargas São Paulo SP Brasil Fundação Getúlio Vargas. São Paulo, SP, Brasil; IX Universidade Federal de Minas Gerais Departamento de Saúde Mental Belo Horizonte MG Brasil Universidade Federal de Minas Gerais. Departamento de Saúde Mental. Belo Horizonte, MG, Brasil; X Universidade Federal de São Paulo Programa de Pós-Graduação em Medicina São Paulo SP Brasil Universidade Federal de São Paulo. Programa de Pós-Graduação em Medicina (Otorrinolaringologia). São Paulo, SP, Brasil; XI Universidade Federal de São Paulo Departamento de Pediatria e Psicobiologia São Paulo SP Brasil Universidade Federal de São Paulo. Departamento de Pediatria e Psicobiologia. São Paulo, SP, Brasil; XII Universidade de São Paulo Hospital das Clínicas Faculdade de Medicina São Paulo SP Brasil Universidade de São Paulo. Hospital das Clínicas da Faculdade de Medicina. Instituto do Coração. Unidade de Hipertensão. São Paulo, SP, Brasil; XIII Stockholms Universitet Psykologiska institutionen Stressforskningsinstitutet Stockholm Sverige Stockholms Universitet. Psykologiska institutionen. Stressforskningsinstitutet. Stockholm, Sverige

**Keywords:** Sleep Initiation and Maintenance Disorders, epidemiology, Risk Factors, Socioeconomic Factors, Case-Control Studies, COVID-19

## Abstract

**OBJECTIVE:**

To identify lifestyle-related, sociodemographic, and mental health characteristics of people with insomnia symptoms and people without insomnia during the pandemic.

**METHODS:**

A case-control study was conducted with data collected by snowball sampling using an online questionnaire. From November 2020 to April 2021, 6,360 people with a mean age of 43.5 years (SD = 14.3) participated in the survey. For this study, we considered 158 cases of insomnia disorder and 476 controls (three controls per case) randomly selected from the participants without sleep problems.

**RESULTS:**

The results of the comparative analysis between cases and controls showed that sleeping less than six hours daily (OR = 3.89; 95%CI 2.50–6.05), feeling sadness frequently (OR = 2.95; 95%CI 1.69–5.17), residing in metropolitan areas (OR = 1.71; 95%CI 1.04–2.84), being 40 years or older (OR = 1.93; 95%CI 1.22–3.06), and the interaction between occupation and poorer education (OR = 2.12; 95%CI 1.22–3.69) were predictors for symptoms of insomnia disorder during the pandemic.

**CONCLUSIONS:**

In addition to confirming the hypothesis that mental health problems are associated with insomnia symptoms, the results point to insomnia as an important outcome for studies on the effects of unemployment, vulnerability and low education of the population, especially in large cities, highlighting that the effects of the crisis on health and the economy are extremely unequally distributed.

## INTRODUCTION

The covid-19 pandemic by the SARS-CoV-2 coronavirus has, since the first case reports, presented challenges for maintaining global health. Different social dynamics imposed by the health crisis induced people to confront a series of abrupt changes in their social routines and life habits^1^.

Studies prior to the covid-19 pandemic have already shown that home isolation recommendations or quarantines imposed on people potentially exposed to a contagious disease can cause manifestations of psychological illness, including symptoms of post-traumatic stress disorder, confusion, anxiety, depression, and burnout^2-5^. This condition of social withdrawal can also disrupt sleep, which is known to be associated with mental health^6^.

The pandemic context of covid-19 is already measured as an independent factor affecting sleep in the population, in which the most commonly self-reported complaint is insomnia^7^. Insomnia disorder is characterized by complaints of persistent and frequent difficulty falling asleep, maintaining sleep, frequent awakenings during sleep, or early morning awakenings, which occur despite the opportunity for sleep and reflect in daytime consequences or impairments^8,9^. Besides insomnia being the most recurrent complaint among people with sleep problems, it is also a risk factor for depression^10^.

In a previous study, 70% of the participants reported sleep problems and 80% anxiety symptoms during the pandemic. The most frequently reported symptom was difficulty falling asleep three or more times a week. Considering that sleep is crucial for the morphofunctional and emotional maintenance of the human body, our hypothesis is that people with mental health related problems were more likely to experience symptoms of insomnia during the pandemic^11^. In this context, the aim of this study was to identify lifestyle-related, sociodemographic, and mental health characteristics of people with insomnia symptoms (cases) and people without symptoms (controls) during the covid-19 pandemic. The cases were identified from a previous study^11^.

## METHODS

### Study Population

This is a case-control study nested within a population-based survey^11^ conducted by the Faculdade de Saúde Pública of Universidade de São Paulo, in collaboration with the *Associação Brasileira do Sono* (ABS) and the *Associação Brasileira de Medicina do Sono* (ABMS). Data were collected by snowball sampling via an online questionnaire during the covid-19 pandemic by the SARS-CoV-2 coronavirus. Invitations to participate were sent via the ABS social networks to the general population. Data collection was conducted from November 2020 to April 2021, in which 6,360 participants aged 10 to 87 years were included (mean age 43.5 years; SD = 14.3)^11^. From this database, cases and controls were randomly selected for the present study.

The sample size for this case-control study was calculated assuming an outcome frequency (insomnia symptoms) of 30%^12^, α = 5.0%, (1-β) = 90%, and a replacement rate of 20%. People who reported symptoms of insomnia were considered cases (158 cases). Three controls per case (476 controls) were randomly selected from the participants without sleep problems. No matching was performed between case and control according to education, occupation, age, or gender. This procedure was adopted to meet the objective of identifying the factors that could contribute to increasing the chance of developing insomnia in the sample studied.

The Ethics Committee of the Faculdade de Saúde Pública of the Universidade de São Paulo approved the study (#39395520.8.0000.5421) and participants were informed about its purpose and data use.

### Data Collection and Tabulation

Participants answered questions about quality, duration, and symptoms related to the sleep-wake cycle (including timing) before and during the pandemic. Sociodemographic data on age, gender, education, and occupation were included in the questionnaire, as well as lifestyle data such as alcohol consumption, smoking, and their respective frequency. The nutritional status information was used to calculate the body mass index (BMI). Nutritional status was characterized according to the WHO classification. In addition, the questionnaire included questions related to habits before going to sleep, such as the use of electronic equipment, reading, exercising, among others.

The symptoms of insomnia included difficulty falling asleep, difficulty staying asleep, and waking up early in the morning and not going back to sleep, according to a scale taken from the Karolinska Sleep Questionnaire^12^. Sleep time was accessed by means of an open-ended question, adopting a Likert scale to assess satisfaction with sleep duration. The survey also included questions about the occurrence and frequency of nightmares, snoring, sleep apnea, bruxism, restless leg symptoms, sleepwalking, and napping before and during the pandemic period^12^.

The outcome “insomnia symptoms” was structured from the affirmative response to one of the three conditions that characterize it (difficulty initiating sleep, difficulty maintaining sleep, and/or early morning awakening), with a frequency of three or more times per week^12^. Insomnia symptoms were considered a case when the first episode occurred during the pandemic.

Regarding mental health, the survey included questions about anxiety, depression, and burnout symptoms before and during the pandemic. Questions related to anxiety symptoms included feeling nervous, anxious, or not being able to stop or control worry. These anxiety symptoms were addressed using the Generalized anxiety disorder-2 (*GAD-2*) scale^13^. The questionnaire also included the frequency of depressed mood and anhedonia, addressing little interest or pleasure in doing things and feeling depressed, down or hopeless (Patient health questionnaire-2)^14^. The frequencies of both symptoms were classified into “not at all, several days, more than half of the days, almost every day.” In addition, the questionnaire also included a scale addressing burnout symptoms before and during the pandemic^15^. Chronotype was assessed by the final item of the morning-evening scale^16^, in which the respondent makes a self-assessment of their chronotype. The response was evaluated by the individual score of the respective item.

Participants who were employed, business owners, employers, or self-employed were grouped into the “active worker” category. Retired, student, and unemployed participants were grouped into the “non-working” category. For the analyses regarding occupation and education, the participants were divided into the following groups: active worker/no college degree; active worker/higher education; non-worker/no college degree; non-worker/ higher education.

### Statistical Analysis

The chi-square test of association was used to analyze the factors associated with insomnia. Then, multiple logistic regression analysis was performed, considering the variables with p < 0.200 in the univariate analysis. The risk measure was the odds ratio (OR) and multiple analysis by the stepwise forward method with the descriptive level p < 0.050. The fit of the models was evaluated using the Hosmer-Lemeshow test. The software STATA version 14 was used for statistical analyses.

## RESULTS

The sample consisted of 488 women and 146 men, totaling 634 participants, of whom 158 had symptoms of insomnia. The participants were adults between the ages of 18 and 79 from various regions of Brazil, with a higher concentration of respondents from Rio de Janeiro and São Paulo. There was a statistically significant association between the presence of insomnia symptoms and age group (p = 0.013), education (p = 0.030), occupation (p < 0.001), and State (p = 0.019). Insomnia symptoms were more frequent among older participants, people with less education, and the unemployed (Table 1).

**Table 1:** Distribution of participants according to sociodemographic data and insomnia symptoms.

Variable	Symptoms of insomnia				p^a^

	No		Yes		
	
	n	%	n	%	
Gender identity					
Female	361	74	127	26	0.24
Male	115	78.8	31	21.2	
Age Group					
18 to 30	90	82.6	19	17.4	0.013
30 to 39	133	81.1	31	18.9	
40 to 49	95	66.9	47	33.1	
50 to 59	95	73.1	35	26.9	
60 to 79	63	70.8	26	29.2	
Having children					
No	211	75.1	70	24.9	0.996
Yes	265	75.1	88	24.9	
Education					
High school complete or less	78	67.2	38	32.8	0.03
Incomplete higher education	56	70	24	30	
Higher education complete	129	74.6	44	25.4	
Postgraduate	213	80.4	52	19.6	
Occupation					
Retired	47	66.2	24	33.8	< 0.001
Unemployed	47	58	34	42	
Employed	192	80.7	46	19.3	
Entrepreneur/employer	31	86.1	5	13.9	
Student	42	65.6	22	34.4	
Liberal professional	117	81.3	27	18.8	
State (place of residence)					
São Paulo	149	81.4	34	18.6	0.019
Rio de Janeiro	97	67.8	46	32.2	
Other	230	74.7	78	25.3	
Nutritional status^b^					
Underweight or eutrophic	207	77.5	60	22.5	0.365
Overweight	151	71.9	59	28.1	
Obesity	118	75.6	38	24.4	

Total	476	75.1	158	24.9	

^a^ Chi-square test.

^b^ Total less than 634 due to no response.

The results of the univariate analysis showed that the participants most likely to have symptoms of insomnia were those who slept less than six hours daily (p < 0.001), were dissatisfied with sleep duration before and during the pandemic (p < 0.001), were dissatisfied with sleep quality before and during the pandemic (p < 0.001), experienced excessive daytime sleepiness (p = 0.021), reported sleep apnea (p = 0.012), woke up with a headache (p = 0.001), had nightmares (p = 0.010), felt discomfort in their legs or needed to move their legs at bedtime (p < 0.001), and woke up feeling tired (p < 0.001) (Table 2).

**Table 2:** Distribution of participants according to sleep characteristics and insomnia symptoms.

Variable	Symptoms of insomnia				p^a^

	No		Yes		
	
	n	%	n	%	
Self-perception of chronotype					
Definitely active in the morning	224	77.2	66	22.8	0.508
Somewhat active in the morning	77	76.2	24	23.8	
Definitely active at night	108	73.5	39	26.5	
Somewhat active at night	65	70.7	27	29.3	
No information	2	50	2	50	
Total sleep time during the pandemic^b^					
6 hours or more	394	84	75	16	< 0.001
Less than 6 hours	75	50	75	50	
Satisfaction with sleep duration (≥ 6 hours)^b^					
Had enough sleep before and during	372	85.1	65	14.9	< 0.001
Had insufficient sleep before and got better during	20	66.7	10	33.3	
Had enough sleep before and got worse during	63	52.1	58	47.9	
Had insufficient sleep before and during	12	41.4	17	58.6	
Satisfaction with sleep quality (before and during the pandemic)^b^					
Good quality before and after	320	88.9	40	11.1	< 0.001
Poor quality before and good quality after	31	88.6	4	11.4	
Good quality before and bad quality after	92	56.8	70	43.2	
Poor quality before and after	25	37.9	41	62.1	
Snoring					
No	342	76.2	107	23.8	0.323
Yes	134	72.4	51	27.6	
Excessive daytime sleepiness					
No	339	77.8	97	22.2	0.021
Yes	137	69.2	61	30.8	
Sleep Apnea					
No	432	76.6	132	23.4	0.012
Yes	44	62.9	26	37.1	
Grinding or clenching of teeth during sleep					
No	334	75.7	107	24.3	0.563
Yes	142	73.6	51	26.4	
Waking up with a headache					
No	304	79.6	78	20.4	0.001
Yes	172	68.3	80	31.7	
Nightmares					
No	376	77.5	109	22.5	0.01
Yes	100	67.1	49	32.9	
Leg discomfort or the need to move legs at bedtime					
No	393	80.7	94	19.3	< 0.001
Yes	83	56.5	64	43.5	
Waking up feeling tired					
No	235	87.4	34	12.6	< 0.001
Yes	241	66	124	34	
Sleepwalking					
No	470	92.2	40	154	0.266
Yes	6	13	40	4	
No problem					
No	475	75	158	25	0.564
Yes	1	100	0	0	

Total	476	75.1	158	24.9	

^a^ Chi-square test.

^b^ Total less than 634 due to no response.

All symptoms associated with characteristics of mental health impairment showed statistically significant association with insomnia symptoms (p < 0.001). Participants who reported increased stress or anxiety symptoms during the pandemic had a higher frequency of insomnia symptoms (29.7%) than the others (8.4%).

The frequency of insomnia symptoms was highest among those who reported little interest/enjoyment in doing things and/or feeling sadness/depression/lack of hope at an almost daily frequency (49.6%), followed by those who reported frequency on more than half of the days of the week (30.4%), and lowest among those who reported frequency a few times a week or once in a while (16.0%) or not at all (9.5%).

Insomnia symptoms were present in 52.5% of the participants who complained about nervousness/anxiety on an almost daily basis; 24% of those who reported it on more than half of the days of the week; 17.6% on an occasional basis; and 5.1% of those who reported not having any. The presence or absence of burnout syndrome symptoms also pointed to statistically significant differences in terms of frequency of insomnia symptoms (40.0% versus 17.2%, respectively).

Multiple logistic regression analysis showed that the independent characteristics statistically associated with the presence of insomnia symptoms were: total sleep time less than 6h (OR = 3.89; 95%CI 2.50–6.05), depression symptoms on more than half of the days (OR = 2.95; 95%CI 1.69–5.17) or almost daily (OR = 4.62; 95%CI 2.84–7.53), States of São Paulo and Rio de Janeiro (OR = 1.71; 95%CI 1.04–2.84), age group over 40 years (OR = 0.93; 95%CI 1.22–3.06), and the interaction between occupation and poorer education (retired/unemployed/student and without complete college education) (OR = 2.12; 95%CI 1.22–3.69). The model was controlled for gender, and residual analysis using the Hosmer-Lemeshow test (c^2^= 3.64; p = 0.888) showed good model fit (Figure).

**Figure:**
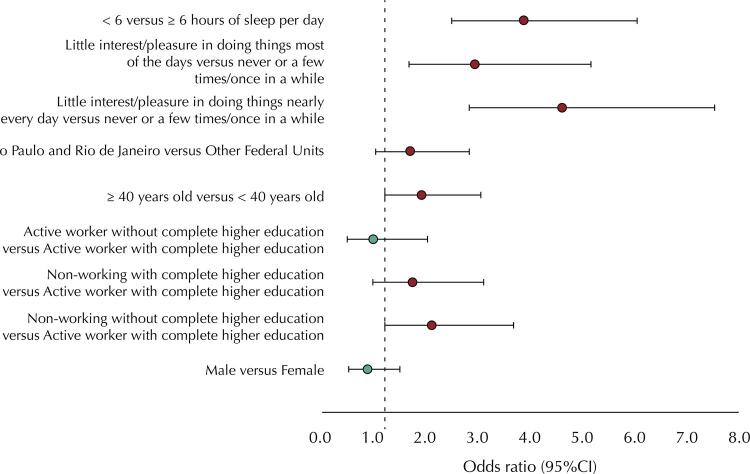
Independent factors associated with insomnia symptoms by multiple regression analysis.

## DISCUSSION

The results of this study showed that sleeping less than six hours a day, feeling sad frequently, residing in a metropolis, being 40 years old or older, and the interaction of occupation and poorer education were predictors for insomnia symptoms during the pandemic. In addition to confirming the hypothesis that mental health symptoms are associated with insomnia symptoms, this study revealed, in an unprecedented way, the interaction between education and occupation as a risk factor for the development of insomnia symptoms. This result was evident in a pandemic scenario, in which people with low education and unemployed were the ones who suffered the most from economic restrictions with the reduction of informal work and, in many cases, the impossibility of obtaining income. In June 2020, for example, the period prior to the present study, the National Household Sample Survey (Pnad) revealed that the level of occupation of the population was around 49% in the first three weeks of the month^17^ .This represents an increase of 1.3 points in the unemployment rate in relation to the quarter before the lockdown decree, reaching a record level of the population outside the labor market^18^.

The increase in job insecurity during the pandemic and its consequent impact on the most vulnerable population, not only from a financial point of view, but also in terms of mental and physical health, including the effects of prejudice and domestic violence, is undeniable^19^. A recent systematic review with 7,744,469 participants from 110,249 different regions of the world demonstrated the association between inadequate income and poor mental health^20^. Considering the association between insomnia and mental health, the present study places insomnia as an important outcome for studies on the effects of unemployment, vulnerability, and low education of the population, especially in large metropolises, emphasizing that the effects of the crisis on health and the economy are extremely unequally distributed^18^.

A recent study conducted in Hong Kong with 3,000 participants^21^ identified that the sociodemographic factors associated with impairment in pandemic-related psychological experience were: middle age, low education, unemployment, low income, and watching pandemic news reports daily for more than one hour. Brooks et al.^3^ identified as stressors longer duration of lockdown, fears about infection, frustration, boredom, inadequate supply, disorientation, financial losses, and stigma. The results of these and the present study reinforce the thesis that prolonged isolation and lack of support exacerbated existing vulnerabilities and contributed to the emergence of stress-related disorders, suggesting that the covid-19 pandemic period should be considered from the perspective of a mass traumatic event^21,22^ Furthermore, these studies point out that, besides the need for public actions to promote financial assistance to vulnerable populations, it is of utmost relevance to offer mental health assistance in health emergencies.

It is worth noting that the increase in mental health problems and insomnia during the pandemic has been evidenced in studies conducted in various countries and population groups. A cutoff study that followed for 10 years the natural course of insomnia in 1434 adults in Canada contacted participants for a survey on insomnia, perceived stress, and other psychological symptoms during the pandemic. The data revealed an incidence of insomnia about four times higher than that reported in a similar interval in the pre-pandemic period^23^. A study in Sweden showed similar results with 45% of people meeting criteria for depression, anxiety, or insomnia^24^. Researchers at the University of Pennsylvania conducted a longitudinal study conducted over 2020 in which they included 3560 participants. The results suggest that concerns about covid-19 were a more consistent predictor of insomnia than exposure to the virus itself^25^. A meta-analysis of 66 studies (n = 221,970) was conducted to assess the prevalence of depression, anxiety, stress, and insomnia, during the pandemic in different population groups^26^. The authors noted that patients with chronic illnesses, people in lockdown, patients with suspected covid, doctors and nurses had a higher prevalence of depression, anxiety, stress and insomnia. In another meta-analysis (68 studies; n = 189,159) the mental health consequences of covid-19 were found to be equally high in all affected countries and for all genders^27^.

Such data indicate that the mode of coping in the face of covid-19 was important in mental health and sleep issues of the population. The association between hopelessness/ sadness/depression and the presence of insomnia symptoms in the present study supports the relevance of this two-way street between mental health and insomnia.

The present study also revealed an increased chance of developing insomnia symptoms starting at age 40. The reduction in sleep duration in this age group corroborates the literature in the area. A US population survey, for example, shows that sleep duration over the life course is U-shaped, i.e., it decreases with aging, reaching a minimum at age 40, and increases around age 50^28^. However, insomnia may not be the only hypothesis for reduced sleep in this age group. In addition, it is important to note that the prevalence of insomnia symptoms in the adult population is associated with conditions of vulnerability, such as low socioeconomic status, poor health status, including physical and psychiatric aspects, and low quality of life^29^.

Psycho-educational programs in crisis situations and/or among populations at high risk of burnout have shown that cultivating positive feelings, behaviors, and thoughts favors emotional resilience, inhibiting the development of symptoms of insomnia, depression, anxiety, post-traumatic stress disorder, and burnout^30^. During the pandemic, programs of this nature demonstrated that careful counseling regarding healthy behaviors associated with strategies for early identification of emotional problems favored improvements in psychological parameters such as resilience, well-being, symptoms of post-traumatic stress disorder, mood, as well as better pandemic management^31^. In Brazil, despite the large contingent of psychologists working at the Unified Health System (SUS) and Reference Centers for Social Assistance (Reference Center for Social Assistance - Cras and Specialized Reference Center for Social Assistance - Creas), approximately 40,000 and 8,000 professionals, respectively, the organization of task forces and the use of digital platforms and telepsychology for intervention and guidance of socioemotional skills among the population have not been adequately planned^32^.

This study has some limitations worth mentioning. Collecting data through social media may have generated the bias of attracting people with sleep problems, somatic symptom disorders, or simply interested in the topic. The use of the data collected for a case-control study sought to mitigate this possible bias. Despite this, there is a predominance of the female gender in the study. It is also important to mention that the sample is not representative of all regions in the country.

On the other hand, the results presented here are important to support actions and public policies for mental health care, especially in health emergencies. In addition, they reinforce the need for screening and early intervention of sleep problems, which are determinants for the health and quality of life of the population and point to the need for measures of attention to mental health and sleep in the post-isolation pandemic phase in order to shorten the suffering of the most vulnerable population.
