# Supplementary material for: Insomnia symptoms during the covid-19 pandemic: a case-control study
Source: Rev Saude Publica. 2023 May 11;57(Suppl 1):8s. doi: 10.11606/s1518-8787.2023057004801 (PMC10185312; doi:10.11606/s1518-8787.2023057004801)
Supplement: Translation

# Translation: Sintomas de insônia durante a pandemia de covid-19: um estudo caso-controle

**Authors:** Andrea Bacelar, Silvia Gonçalves Conway, Márcia Assis, Victor Menezes Silva, Pedro Rodrigues Genta, Daniela Vianna Pachito, Almir Ribeiro Tavares, Danilo Anunciatto Sguillar, Gustavo Antonio Moreira, Luciano Ferreira Drager, Claudia Roberta de Castro Moreno

**Affiliations:** I Brasil Clínica Neurológica Carlos Bacelar. Rio de Janeiro, RJ, Brasil; II Brasil Associação Brasileira do Sono. São Paulo, SP, Brasil; III Brasil Universidade de São Paulo. Faculdade de Medicina. Departamento de Psiquiatria. São Paulo, SP, Brasil; IV Brasil Akasa - Formação e Conhecimento. São Paulo, SP, Brasil; V Brasil Hospital São Lucas. Clínica do Sono de Curitiba. Curitiba, PR, Brasil; VI Brasil Universidade de São Paulo. Faculdade de Saúde Pública. Departamento de Saúde, Ciclos de Vida e Sociedade.São Paulo, SP, Brasil; VII Brasil Universidade de São Paulo. Hospital das Clínicas da Faculdade de Medicina. Instituto do Coração. Laboratório do Sono. São Paulo, SP, Brasil; VIII Brasil Fundação Getúlio Vargas. São Paulo, SP, Brasil; IX Brasil Universidade Federal de Minas Gerais. Departamento de Saúde Mental. Belo Horizonte, MG, Brasil; X Brasil Universidade Federal de São Paulo. Programa de Pós-Graduação em Medicina (Otorrinolaringologia). São Paulo, SP, Brasil; XI Brasil Universidade Federal de São Paulo. Departamento de Pediatria e Psicobiologia. São Paulo, SP, Brasil; XII Brasil Universidade de São Paulo. Hospital das Clínicas da Faculdade de Medicina. Instituto do Coração. Unidade de Hipertensão. São Paulo, SP, Brasil; XIII Sverige Stockholms Universitet. Psykologiska institutionen. Stressforskningsinstitutet. Stockholm, Sverige

**Keywords:** Distúrbios do Início e da Manutenção do Sono, epidemiologia, Fatores de Risco, Fatores Socioeconômicos, Estudos de Casos e Controles, COVID-19

## Abstract

**OBJETIVO:**

Identificar características relacionadas ao estilo de vida, sociodemográficas e saúde mental de pessoas com sintomas de insônia e pessoas sem insônia durante a pandemia.

**MÉTODOS:**

A partir de dados coletados por amostragem em bola de neve, por meio de um questionário online foi realizado um estudo caso-controle. Durante o período de novembro de 2020 a abril de 2021, 6.360 pessoas com idade média de 43,5 anos (DP = 14,3) participaram da pesquisa. No presente estudo, foram considerados 158 casos de transtorno de insônia e 476 controles (três controles por caso) selecionados aleatoriamente dentre os participantes sem problemas de sono.

**RESULTADOS:**

Os resultados da análise comparativa entre casos e controles mostraram que dormir menos de seis horas diárias (OR = 3,89; IC95% 2,50–6,05), sentir tristeza frequentemente (OR = 2,95; IC95% 1,69–5,17), residir em metrópoles (OR = 1,71; IC95% 1,04–2,84), estar com 40 anos ou mais (OR = 1,93; IC95% 1,22–3,06) e a interação entre ocupação e escolaridade mais precária (OR = 2,12; IC95% 1,22–3,69) foram fatores preditores para sintomas de transtorno de insônia durante a pandemia.

**CONCLUSÕES:**

Além da confirmação da hipótese de que problemas de saúde mental estão associados a sintomas de insônia, os resultados apontam para a insônia como um desfecho importante para estudos sobre efeitos do desemprego, vulnerabilidade e baixa escolaridade da população, sobretudo nas grandes metrópoles, ressaltando que os efeitos da crise sobre a saúde e a economia são distribuídos de forma extremamente desiguais.

## INTRODUÇÃO

A pandemia de covid-19 pelo coronavírus SARS-CoV-2 apresentou, desde as primeiras notificações de casos, desafios para a manutenção da saúde global. Diferentes dinâmicas sociais, impostas pela condição sanitária, induziram as pessoas a confrontarem uma série de abruptas mudanças em suas rotinas sociais e hábitos de vida^1^.

Estudos anteriores à pandemia de covid-19 já demostravam que situações de recomendações de isolamento domiciliar ou quarentenas impostas a pessoas potencialmente expostas a alguma doença contagiosa podem provocar manifestações de adoecimento psicológico, incluindo sintomas de transtorno de estresse pós-traumático, confusão, ansiedade, depressão e esgotamento^2^. Essa condição de distanciamento social também pode perturbar o sono, que é sabidamente associado à saúde mental^6^.

O contexto pandêmico de covid-19 já é mensurado enquanto fator independente que afeta o sono da população, na qual a queixa mais autorreferida é a insônia^7^. O transtorno de insônia é caracterizado por queixas de dificuldade persistente e frequente em adormecer, manter o sono, despertares frequentes durante o sono ou despertares matinais precoces, que acontecem apesar da oportunidade para dormir e refletem em consequências ou comprometimentos diurnos^8,9^. Além de a insônia ser a queixa mais recorrente entre as pessoas com problemas de sono, também é um fator de risco para depressão^10^.

Em um estudo prévio, 70% dos participantes relataram problemas de sono e 80%, sintomas de ansiedade durante a pandemia. O sintoma mais frequentemente relatado foi a dificuldade para adormecer três ou mais vezes por semana. Considerando que o sono é crucial para a manutenção morfofuncional e emocional do corpo humano, nossa hipótese é que as pessoas com problemas relacionados à saúde mental estavam mais propensas a apresentar sintomas de insônia durante a pandemia^11^. Nesse contexto, o objetivo deste estudo foi identificar características relacionadas ao estilo de vida, sociodemográficas e de saúde mental de pessoas com sintomas de insônia (casos) e pessoas sem sintomas (controles) durante a pandemia de covid-19. Os casos foram identificados a partir de estudo anterior^11^.

## MÉTODOS

### População do Estudo

Este é um estudo caso-controle aninhado a um inquérito populacional^11^ realizado pela Faculdade de Saúde Pública da Universidade de São Paulo, em colaboração com a Associação Brasileira do Sono (ABS) e a Associação Brasileira de Medicina do Sono (ABMS). Os dados foram coletados por amostragem em bola de neve, por meio de um questionário online durante a pandemia de covid-19 pelo coronavírus SARS-CoV-2. O convite para participação foi enviado pelas redes sociais da ABS para a população em geral. A coleta de dados foi realizada de novembro de 2020 a abril de 2021, na qual foram incluídos 6.360 participantes com idades entre 10 e 87 anos (idade média de 43,5 anos; DP = 14,3)^11^. A partir dessa base de dados, casos e controles foram aleatoriamente selecionados para a realização do presente estudo.

O tamanho da amostra deste estudo caso-controle foi calculado assumindo a frequência de desfecho (sintomas de insônia) de 30%^12^, α = 5,0%, (1-β) = 90% e taxa de reposição de 20%. As pessoas que relataram sintomas de insônia foram consideradas casos (158 casos). Três controles por caso (476 controles) foram selecionados aleatoriamente dentre os participantes sem problemas de sono. Não foi realizado pareamento entre caso e controle segundo escolaridade, ocupação, idade ou gênero. Esse procedimento foi adotado para atender o objetivo de identificar os fatores que poderiam contribuir para aumentar a chance de desenvolvimento de insônia na amostra estudada.

O Comitê de Ética da Faculdade de Saúde Pública da Universidade de São Paulo aprovou o estudo (#39395520.8.0000.5421) e os participantes foram informados sobre seu objetivo e uso de dados.

### Coleta e Tabulação dos Dados

Os participantes responderam questões sobre qualidade, duração e sintomas relacionados ao ciclo vigília-sono (incluindo alocação temporal) antes e durante a pandemia. Dados sociodemográficos referentes à idade, gênero, escolaridade e ocupação foram incluídos no questionário, além de dados sobre estilo de vida como consumo de álcool, tabagismo e sua respectiva frequência. As informações do estado nutricional foram utilizadas para calcular o índice de massa corporal (IMC). O estado nutricional foi caracterizado de acordo com a classificação da OMS. Além disso, o questionário incluiu questões relacionadas aos hábitos antes de dormir, como uso de equipamentos eletrônicos, leitura, prática de exercícios, dentre outros.

Os sintomas de insônia incluíam dificuldade em adormecer, dificuldade em manter o sono e acordar cedo pela manhã e não voltar a dormir, conforme escala extraída do questionário de sono de Karolinska (*Karolinska Sleep Questionnaire)*^12^. O tempo de sono foi acessado por meio de uma questão aberta, adotando-se uma escala Likert para avaliar a satisfação com a duração do sono. A pesquisa também incluiu questões sobre a ocorrência e a frequência de pesadelos, roncos, apneia do sono, bruxismo, sintomas de pernas inquietas, sonambulismo e cochilos antes e durante o período de pandemia^12^.

O desfecho “sintomas de insônia” foi estruturado a partir da resposta afirmativa a uma das três condições que a caracterizam (dificuldade em iniciar o sono, dificuldade em manter o sono e/ou despertar precoce matutino), com frequência de três ou mais vezes por semana^12^. Considerou-se caso de sintomas de insônia quando o primeiro episódio ocorreu durante a pandemia.

Em relação à saúde mental, a pesquisa incluiu perguntas sobre ansiedade, depressão e sintomas de *burnout* antes e durante a pandemia. As questões relacionadas aos sintomas de ansiedade incluíram sentir-se nervoso, ansioso ou não conseguir parar ou controlar a preocupação. Esses sintomas de ansiedade foram abordados por meio da escala de Transtorno de Ansiedade Generalizada-2 (*Generalized anxiety disorder-2 – GAD-2*)^13^. O questionário também incluiu a frequência de humor deprimido e anedonia, abordando pouco interesse ou prazer em fazer as coisas e sentir-se deprimido, deprimido ou sem esperança (*Patient health questionnaire-2*)^14^. As frequências de ambos os sintomas foram classificadas em “nada, vários dias, mais da metade dos dias, quase todos os dias”. Além disso, o questionário também incluiu uma escala abordando sintomas de *burnout* antes e durante a pandemia^15^. O cronotipo foi avaliado pelo item final da escala de matutinidade-vespertinidade^16^, no qual o respondente faz uma autoavaliação de seu cronotipo. A resposta foi avaliada pela pontuação individual do respectivo item.

Os participantes empregados, empresários, empregadores ou profissionais liberais foram agrupados na categoria “trabalhador ativo”. Já os participantes aposentados, estudantes e desempregados foram agrupados na categoria “não trabalhador”. Para as análises relativas à ocupação e escolaridade, os participantes foram divididos nos seguintes grupos: trabalhador ativo/sem curso superior; trabalhador ativo/com curso superior; não trabalhador/sem curso superior; não trabalhador/com curso superior.

### Análises Estatísticas

Para análise dos fatores associados à insônia foi utilizado o teste de associação pelo qui-quadrado. Em seguida, foi realizada análise de regressão logística múltipla, considerando as variáveis com p < 0,200 na análise univariada. A medida de risco foi o odds ratio (OR) e a análise múltipla pelo método *stepwise forward* com o nível de descritivo p < 0,050. O ajuste dos modelos foi avaliado por meio do teste de Hosmer-Lemeshow. Para realização das análises estatísticas foi utilizado o software STATA versão 14.

## RESULTADOS

A amostra foi constituída por 488 mulheres e 146 homens, totalizando 634 participantes, dos quais 158 apresentavam sintomas de insônia. Os participantes eram adultos de 18 a 79 anos de diversas regiões do Brasil, com uma concentração maior de respondentes do Rio de Janeiro e São Paulo. Houve associação estatisticamente significativa entre presença de sintomas de insônia e faixa etária (p = 0,013), escolaridade (p = 0,030), ocupação (p < 0,001) e Unidade Federativa (p = 0,019). Os sintomas de insônia foram mais frequentes entre os participantes mais velhos, pessoas de menor escolaridade e em desempregados (Tabela 1).

**Tabela 1:** Distribuição dos participantes segundo dados sociodemográficos e sintomas de insônia.

Variável	Sintomas de insônia				p^a^

	Não		Sim		
	
	n	%	n	%	
Identidade de gênero					
Feminino	361	74	127	26	0,24
Masculino	115	78,8	31	21,2	
Faixa etária					
18 a 30	90	82,6	19	17,4	0,013
30 a 39	133	81,1	31	18,9	
40 a 49	95	66,9	47	33,1	
50 a 59	95	73,1	35	26,9	
60 a 79	63	70,8	26	29,2	
Ter filhos					
Não	211	75,1	70	24,9	0,996
Sim	265	75,1	88	24,9	
Escolaridade					
Ensino médio completo ou menos	78	67,2	38	32,8	0,03
Ensino superior incompleto	56	70	24	30	
Ensino superior completo	129	74,6	44	25,4	
Pós-graduado	213	80,4	52	19,6	
Ocupação					
Aposentado(a)	47	66,2	24	33,8	< 0,001
Desempregado(a)	47	58	34	42	
Empregado(a)	192	80,7	46	19,3	
Empresário(a)/empregador(a)	31	86,1	5	13,9	
Estudante	42	65,6	22	34,4	
Profissional liberal	117	81,3	27	18,8	
Estado (local de residência)					
São Paulo	149	81,4	34	18,6	0,019
Rio de Janeiro	97	67,8	46	32,2	
Demais	230	74,7	78	25,3	
Estado nutricional^b^					
Baixo peso ou eutrofia	207	77,5	60	22,5	0,365
Sobrepeso	151	71,9	59	28,1	
Obesidade	118	75,6	38	24,4	

Total	476	75,1	158	24,9	

^a^ Teste qui-quadrado.

^b^ Total inferior a 634 devido à ausência de resposta.

Os resultados da análise univariada evidenciaram que os participantes com maior chance de apresentarem sintomas de insônia foram os que dormiam menos de seis horas diárias (p < 0,001), estavam insatisfeitos com a duração do sono antes e durante a pandemia (p < 0,001), estavam insatisfeitos com a qualidade do sono antes e durante a pandemia (p < 0,001), sentiam sonolência excessiva diurna (p = 0,021), relatavam apneia do sono (p = 0,012), acordavam com dor de cabeça (p=0,001), tinham pesadelos (p = 0,010), sentiam desconforto nas pernas ou necessidade de mover as pernas na hora de dormir (p < 0,001), e acordavam sentindo-se cansados (p < 0,001) (Tabela 2).

**Tabela 2:** Distribuição dos participantes segundo características do sono e sintomas de insônia.

Variável	Sintomas de insônia				p^a^

	Não		Sim		
	
	n	%	n	%	
Autopercepção de cronotipo					
Definitivamente ativo de manhã	224	77,2	66	22,8	0,508
Um pouco ativo de manhã	77	76,2	24	23,8	
Definitivamente ativo à noite	108	73,5	39	26,5	
Um pouco ativo à noite	65	70,7	27	29,3	
Sem informação	2	50	2	50	
Tempo total de sono durante a pandemia^b^					
6 horas ou mais	394	84	75	16	< 0,001
Menos de 6 horas	75	50	75	50	
Satisfação com a duração do sono (≥ 6 horas)^b^					
Dormia suficiente antes e durante	372	85,1	65	14,9	< 0,001
Dormia insuficientemente antes e melhorou durante	20	66,7	10	33,3	
Dormia suficientemente antes e piorou durante	63	52,1	58	47,9	
Dormia insuficientemente antes e durante	12	41,4	17	58,6	
Satisfação com a qualidade do sono (antes e durante a pandemia)^b^					
Boa qualidade antes e depois	320	88,9	40	11,1	< 0,001
Má qualidade antes e boa qualidade depois	31	88,6	4	11,4	
Boa qualidade antes e má qualidade depois	92	56,8	70	43,2	
Má qualidade antes e depois	25	37,9	41	62,1	
Ronco					
Não	342	76,2	107	23,8	0,323
Sim	134	72,4	51	27,6	
Sonolência excessiva diurna					
Não	339	77,8	97	22,2	0,021
Sim	137	69,2	61	30,8	
Apneia do sono					
Não	432	76,6	132	23,4	0,012
Sim	44	62,9	26	37,1	
Ranger ou apertar os dentes durante o sono					
Não	334	75,7	107	24,3	0,563
Sim	142	73,6	51	26,4	
Acordar com dor de cabeça					
Não	304	79,6	78	20,4	0,001
Sim	172	68,3	80	31,7	
Pesadelos					
Não	376	77,5	109	22,5	0,01
Sim	100	67,1	49	32,9	
Desconforto nas pernas ou necessidade de mover as pernas na hora de dormir					
Não	393	80,7	94	19,3	< 0,001
Sim	83	56,5	64	43,5	
Acordar sentindo-se cansado					
Não	235	87,4	34	12,6	< 0,001
Sim	241	66	124	34	
Sonambulismo					
Não	470	92,2	40	154	0,266
Sim	6	13	40	4	
Nenhum problema					
Não	475	75	158	25	0,564
Sim	1	100	0	0	

Total	476	75,1	158	24,9	

^a^ Teste qui-quadrado.

^b^ Total inferior a 634 devido à ausência de resposta.

Todos os sintomas associados a características de prejuízo da saúde mental evidenciaram associação estatisticamente significativa com sintomas de insônia (p < 0,001). Os participantes que relataram aumento dos sintomas de estresse ou ansiedade durante a pandemia apresentaram maior frequência de sintomas de insônia (29,7%) do que os demais (8,4%).

A frequência dos sintomas de insônia foi mais elevada entre as pessoas que relataram pouco interesse/prazer em fazer coisas e/ou sentimento tristeza/depressão/falta de esperança numa frequência quase diária (49,6%), seguida por aquelas que apresentaram frequência em mais da metade dos dias da semana (30,4%), e mais baixa entre aqueles que reportaram frequência de poucas vezes por semana ou de vez em quando (16,0%) ou nenhuma vez (9,5%).

Os sintomas de insônia estiveram presentes em 52,5% dos participantes que manifestaram queixa de nervosismo/ansiedade numa frequência quase diária; 24% dos que reportaram em mais da metade dos dias da semana; 17,6% em frequência eventual e 5,1% dos que afirmaram não terem apresentado a queixa nenhuma vez. A presença ou ausência de sintomas de síndrome de *burnout* também apontou para diferenças estatisticamente significantes em termos da frequência de sintomas de insônia (40,0% *versus* 17,2%, respectivamente).

A análise de regressão logística múltipla mostrou que as características independentes associadas estatisticamente à presença de sintomas de insônia foram: tempo total de sono inferior a 6 h (OR = 3,89; IC95% 2,50–6,05), sintomas de depressão em mais da metade dos dias (OR = 2,95; IC95% 1,69–5,17) ou quase diária (OR = 4,62; IC95% 2,84–7,53), Unidades Federativas de São Paulo e Rio de Janeiro (OR=1,71; IC95% 1,04–2,84), faixa etária a partir de 40 anos (OR = 0,93; IC95% 1,22–3,06), e a interação entre ocupação e escolaridade mais precária (aposentado/desempregado/estudante e sem Ensino Superior completo) (OR=2,12; IC95% 1,22–3,69). O modelo foi controlado por gênero, e a análise de resíduos por meio do teste de Hosmer-Lemeshow (c^2^= 3,64; p = 0,888) mostrou bom ajuste do modelo (Figura).

**Figura:** Fatores independentes associados à presença de sintomas de insônia por meio da análise de regressão múltipla.

## DISCUSSÃO

Os resultados desse estudo mostraram que dormir menos de seis horas diárias, sentir tristeza frequentemente, residir em metrópoles, estar com 40 anos ou mais e a interação entre ocupação e escolaridade mais precária foram fatores preditores para sintomas de insônia durante a pandemia. Além da confirmação da hipótese de que os sintomas de saúde mental estão associados a sintomas de insônia, o estudo revelou, de forma inédita, a interação entre escolaridade e ocupação como fator de risco para o desenvolvimento de sintomas de insônia. Esse resultado ficou evidente em um cenário pandêmico, no qual as pessoas com baixa escolaridade e desempregadas foram as que mais sofreram restrições econômicas com a redução do trabalho informal e, em muitos casos, a impossibilidade de obtenção de renda. Já em junho de 2020, por exemplo, período anterior à presente pesquisa, a Pesquisa Nacional por Amostra de Domicílios (Pnad) revelava que o nível de ocupação da população estava em torno de 49% nas três primeiras semanas do mês^17^, representando um aumento de 1,3 pontos na taxa de desocupação em relação ao trimestre anterior ao decreto de quarentena, atingindo um recorde da população fora do mercado de trabalho^18^.

É inegável o incremento da precarização do trabalho durante a pandemia e seu consequente impacto na população mais vulnerável, não somente do ponto de vista financeiro, como também da saúde mental e física, incluindo efeitos de preconceito e violência doméstica^19^. Uma recente revisão sistemática reunindo 7.744.469 participantes de 110.249 distintas regiões do planeta demonstrou a associação entre renda inadequada e baixa saúde mental^20^. Considerando a associação entre insônia e saúde mental, o presente estudo coloca a insônia como um desfecho importante para estudos sobre efeitos do desemprego, vulnerabilidade e baixa escolaridade da população, sobretudo nas grandes metrópoles, ressaltando que os efeitos da crise sobre a saúde e a economia são distribuídos de forma extremamente desigual^18^.

Um estudo recente conduzido em Hong Kong com 3.000 participantes^21^ identificou que os fatores sociodemográficos associados ao prejuízo na experiência psicológica relacionada à pandemia foram: meia idade, baixa escolaridade, desemprego, baixa renda e assistir diariamente a reportagens sobre pandemia por mais de uma hora. Brooks et al.^3^ identificaram como fatores estressantes uma maior duração da quarentena, temores acerca da infecção, frustração, tédio, abastecimento inadequado, desorientação, perdas financeiras e estigma. Os resultados destes e do presente estudo reforçam a tese de que o isolamento prolongado e a falta de apoio exacerbaram as vulnerabilidades existentes e contribuíram para o surgimento de transtornos relacionados ao estresse, sugerindo que o período de pandemia de covid-19 deva ser considerado sob a perspectiva de evento traumático em massa^21,22^. Ademais, esses estudos apontam que, além da necessidade de ações públicas para promover a assistência financeira às populações vulneráveis, é de extrema relevância oferecer assistência à saúde mental em emergências sanitárias.

Vale ressaltar que o aumento de problemas de saúde mental e de insônia durante a pandemia foi evidenciado em estudos realizados em diversos países e grupos populacionais. Um estudo de corte que acompanhava por 10 anos o curso natural da insônia em 1434 adultos do Canadá contatou os participantes para uma pesquisa sobre insônia, estresse percebido e outros sintomas psicológicos durante a pandemia. Os dados revelaram a incidência de insônia cerca de quatro vezes maior do que a relatada num intervalo semelhante no período pré-pandêmico^23^. Estudo realizado na Suécia mostrou resultados semelhantes com 45% das pessoas preenchendo critérios para depressão, ansiedade ou insônia^24^. Pesquisadores da Universidade da Pensilvânia realizaram um estudo longitudinal conduzido ao longo de 2020, no qual incluíram 3560 participantes. Os resultados sugerem que preocupações com a covid-19 constituíram um fator preditor de insônia mais consistente do que a exposição ao vírus propriamente dita^25^. Uma metanálise com 66 estudos (n = 221.970) foi realizada para avaliar a prevalência de depressão, de ansiedade, do estresse e da insônia, durante a pandemia em diferentes grupos populacionais^26^. Os autores observaram que pacientes com doenças crônicas, pessoas em quarentena, pacientes com suspeita de covid, médicos e enfermeiras tiveram uma maior prevalência de depressão, ansiedade, estresse e insônia. Em outra metanálise (68 estudos; n = 189,159) observou-se que as consequências da covid-19 para a saúde mental são igualmente altas em todos os países afetados e para todos os gêneros^27^.

Tais dados indicam que o modo de enfrentamento diante da covid-19 foi importante em questões da saúde mental e sono da população. A associação entre a falta de esperança/tristeza/depressão e a presença de sintomas de insônia no presente estudo corrobora a relevância desta via de mão dupla entre a saúde mental e a insônia.

O presente estudo também revelou um aumento da chance de desenvolvimento de sintomas de insônia a partir dos 40 anos. A redução da duração de sono nessa faixa etária corrobora a literatura da área. Um levantamento populacional norte-americano, por exemplo, mostra que a duração do sono ao longo do curso da vida tem uma forma de U, isto é, diminui com o envelhecimento, atingindo um mínimo aos 40 anos, e aumenta por volta dos 50 anos^28^. Entretanto, a insônia pode não ser a única hipótese para a redução do sono nessa faixa etária. Além disso, é importante ressaltar que a prevalência de sintomas de insônia na população adulta está associada a condições de vulnerabilidade, como baixo nível socioeconômico, pior condição de saúde, incluindo aspectos físicos e psiquiátricos e baixa qualidade de vida^29^.

Programas psico-educacionais em situações de crise e/ou entre populações com risco elevado de *burnout* demonstraram que o cultivo de sentimentos, comportamentos e pensamentos positivos favorece a resiliência emocional, inibindo o desenvolvimento de sintomas de insônia, depressão, ansiedade, transtorno de estresse pós-traumático e *burnout*^30^. Durante a pandemia, programas dessa natureza demonstraram que a orientação cuidadosa relativa a comportamentos saudáveis associada a estratégias para identificação precoce de problemas emocionais favoreceu melhoras em parâmetros psicológicos, tais como resiliência, bem-estar, sintomas de transtorno de estresse pós-traumático, humor, assim como melhor manejo da pandemia^31^. No Brasil, apesar do grande contingente de psicólogos atuando junto ao Sistema Único de Saúde (SUS) e Centros de Referência para Assistência Social (Centro de Referência de Assistência Social – Cras e Centro de Referência Especializado de Assistência Social – Creas), aproximadamente 40.000 e 8.000 profissionais, respectivamente, a organização de força tarefa e uso de plataformas digitais e telepsicologia para intervenção e orientação de habilidades socioemocionais junto à população não foram adequadamente planejados^32^.

Este estudo tem algumas limitações que devem ser mencionadas. A coleta de dados por meio de mídia social pode ter gerado o viés de atrair pessoas com problemas de sono, transtornos de sintomas somáticos ou simplesmente interessadas no tema. O uso dos dados coletados para um estudo caso-controle buscou mitigar esse possível viés. Apesar disso, há uma predominância do gênero feminino no estudo. Importante mencionar também que a amostra não é representativa de todas as regiões do país.

Por outro lado, os resultados aqui apresentados são importantes para embasar ações e políticas públicas de atenção à saúde mental, sobretudo em emergências sanitárias. Além disso, reforçam a necessidade do rastreamento e intervenção precoce dos problemas de sono, que são determinantes para saúde e qualidade de vida da população e apontam para a necessidade de medidas de atenção à saúde mental e ao sono na fase pós-isolamento pandêmico a fim de abreviar o sofrimento da população mais vulnerável.
